# Stroke severity mediates the effect of socioeconomic disadvantage on poor outcomes among patients with intracerebral hemorrhage

**DOI:** 10.3389/fneur.2023.1176924

**Published:** 2023-06-13

**Authors:** Thomas B. H. Potter, Jonika Tannous, Alan P. Pan, Abdulaziz Bako, Carnayla Johnson, Eman Baig, Hannah Kelly, Charles D. McCane, Tanu Garg, Rajan Gadhia, Vivek Misra, John Volpi, Gavin Britz, David Chiu, Farhaan S. Vahidy

**Affiliations:** ^1^Department of Neurosurgery, Houston Methodist, Houston, TX, United States; ^2^Center for Health Data Science and Analytics, Houston Methodist, Houston, TX, United States; ^3^Department of Neurology, Houston Methodist, Houston, TX, United States; ^4^Department of Neurology, Weill Cornell Medicine, White Plains, NY, United States; ^5^Department of Neurology, Houston Methodist Academic Institute, Houston Methodist, Houston, TX, United States; ^6^Department of Neurological Surgery, Houston Methodist Neurological Institute, Houston Methodist, Houston, TX, United States; ^7^Department of Population Health Sciences, Weill Cornell Medicine, White Plains, NY, United States

**Keywords:** intracerebral hemorrhagic stroke, socioeconomic disadvantage, cerebral small vessel disease, mediation analysis, patient outcomes

## Abstract

**Background:**

Socioeconomic deprivation drives poor functional outcomes after intracerebral hemorrhage (ICH). Stroke severity and background cerebral small vessel disease (CSVD) burden have each been linked to socioeconomic status and independently contribute to worse outcomes after ICH, providing distinct, plausible pathways for the effects of deprivation. We investigate whether admission stroke severity or cerebral small vessel disease (CSVD) mediates the effect of socioeconomic deprivation on 90-day functional outcomes.

**Methods:**

Electronic medical record data, including demographics, treatments, comorbidities, and physiological data, were analyzed. CSVD burden was graded from 0 to 4, with severe CSVD categorized as ≥3. High deprivation was assessed for patients in the top 30% of state-level area deprivation index scores. Severe disability or death was defined as a 90-day modified Rankin Scale score of 4–6. Stroke severity (NIH stroke scale (NIHSS)) was classified as: none (0), minor (1–4), moderate (5–15), moderate–severe (16–20), and severe (21+). Univariate and multivariate associations with severe disability or death were determined, with mediation evaluated through structural equation modelling.

**Results:**

A total of 677 patients were included (46.8% female; 43.9% White, 27.0% Black, 20.7% Hispanic, 6.1% Asian, 2.4% Other). In univariable modelling, high deprivation (odds ratio: 1.54; 95% confidence interval: [1.06–2.23]; *p* = 0.024), severe CSVD (2.14 [1.42–3.21]; *p* < 0.001), moderate (8.03 [2.76–17.15]; p < 0.001), moderate–severe (32.79 [11.52–93.29]; *p* < 0.001), and severe stroke (104.19 [37.66–288.12]; *p* < 0.001) were associated with severe disability or death. In multivariable modelling, severe CSVD (3.42 [1.75–6.69]; *p* < 0.001) and moderate (5.84 [2.27–15.01], *p* < 0.001), moderate–severe (27.59 [7.34–103.69], *p* < 0.001), and severe stroke (36.41 [9.90–133.85]; *p* < 0.001) independently increased odds of severe disability or death; high deprivation did not. Stroke severity mediated 94.1% of deprivation’s effect on severe disability or death (*p* = 0.005), while CSVD accounted for 4.9% (*p* = 0.524).

**Conclusion:**

CSVD contributed to poor functional outcome independent of socioeconomic deprivation, while stroke severity mediated the effects of deprivation. Improving awareness and trust among disadvantaged communities may reduce admission stroke severity and improve outcomes.

## 1. Introduction

Functional recovery after Intracerebral Hemorrhage (ICH) is characteristically poor, with fewer than 1 in 3 patients expected to achieve premorbid levels of functional independence (1). As limited treatment options are available, it is critical to address the factors and mechanisms that contribute to poor patient outcomes. There is emerging evidence that poor socioeconomic status may independently influence poor ICH outcomes, even when controlled for traditional demographic and clinical factors (2, 3). Concordantly, socioeconomic deprivation has been independently associated with both stroke severity (4) and the vascular risk factors that underly Cerebral Small Vessel Disease (CSVD) (5).

Stroke severity is almost ubiquitously measured via the National Institutes of Health Stroke Scale (NIHSS) (6). The NIHSS is a reliable tool for monitoring ICH patients that assesses neurological function and incident stroke severity (7), and admission NIHSS scores correlate well with post-ICH patient outcomes (8, 9). Separately, functional and cognitive outcomes are also influenced by the background burden of CSVD (10, 11), a subclinical syndrome marked by cerebral lesions from various etiologies that represents cumulative cerebral vascular damage (12–15). Environmental factors, including socioeconomic status, have linked to the risk of CSVD development (5).

Both CSVD and NIHSS-measured stroke severity impact functional recovery and have been associated with socioeconomic status. Each thereby provides a plausible mechanism for the evident effects of socioeconomic deprivation in ICH (11, 16). There is little direct evidence to provide clear support or a cohesive model for either, however. This study seeks to clarify potential linkages between CSVD, stroke severity, and socioeconomic deprivation on 90-day functional outcome among ICH patients.

## 2. Methods

### 2.1. Study protocols, data extraction, and population

The study protocol was approved by the Houston Methodist Institutional Review Board as a minimum-risk study. Data relating to patient hospital encounters were extracted from Registry of Neurological Endpoint Assessments among Patients with Ischemic and Hemorrhagic Stroke (REINAH), an electronic medical record-based registry of patients with cerebrovascular disease (17). REINAH has been established as a comprehensive data resource for primary stroke encounters occurring after May 2016 across the Houston Methodist hospital system, a tertiary healthcare system that includes 7 certified stroke centers and serves the diverse population of ~7.2 million within the Houston Metropolitan Statistical Area (18). Patients with primary stroke encounters are selected for REINAH inclusion if they have documented *International Statistical Classification of Diseases, Tenth Revision* (ICD-10) discharge diagnosis codes of acute ischemic stroke (ICD-10: I63), non-traumatic ICH (ICD-10: I61) transient ischemic attack (ICD: G45), or subarachnoid hemorrhage (ICD: I60). Patient outcomes were obtained from the Hospital Outcomes-based Prospective Endpoints in Stroke registry, which records the treatment metrics and characteristics of acute ischemic stroke, ICH, transient ischemic attack, and subarachnoid hemorrhage patients, and seeks to collect 90-day functional outcome via telephone assessment (19).

The population of interest for this study were adult patients (>18 years of age at encounter) with primary spontaneous ICH. Patients were included in this study if they received a primary discharge diagnosis of ICH (ICD-10: I61.0-I61.9). Patients were excluded from study if they had missing or incomplete hemorrhage characteristics, exhibited secondary or traumatic ICH, did not have available address information, or did not have 90-day functional outcome assessment.

### 2.2. Clinical and imaging variables and outcomes

Data extracted from the REINAH included demographic information, comorbidities, hospital treatment metrics, and measures of stroke severity. The primary exposure of interest was socioeconomic deprivation, measured for patients using the state-level Area Deprivation Index (ADI) (20, 21). Briefly, the ADI is an aggregate measure for 17 distinct metrics that reflect the degree of socioeconomic disadvantage, including measures of income and wealth, property ownership, access (telephone, car, etc.), and crowding (20, 22). Patient ADI measures were determined based on exact residential addresses. The ADI was analyzed as a decile rank, with higher rank representing greater neighborhood deprivation. Patients in the top 30% of state-level ADI distribution (ADI ≥ 8) were classified as “High deprivation” (HD). The primary outcome of interest was severe disability or death (SDD), defined as a 90-day modified Rankin Scale (mRS) score of 4–6.

Hemorrhage characteristics were recorded for each patient based on the first computed tomography (CT) scan collected as a part of their primary ICH encounter. Hemorrhage volumes were manually assessed using the ABC/2 method and were recorded along with hemorrhage location, laterality, and the presence of intraventricular or extra-axial hemorrhage. Magnetic Resonance Imaging (MRI) images were assessed for markers of CSVD, including Fazekas-scored white matter hyperintensities in the deep and periventricular white matter, number of cerebral microbleeds, number of lacunes, and scored enlarged perivascular spaces (ePVS) (16). Data from these CSVD markers were aggregated into a single CSVD score (0–4), where 1 point was assigned for each of the following markers: (1) deep white matter hyperintensity score of 2–3 or periventricular white matter hyperintensity score of 3; (2) presence of any microbleed; (3) presence of any lacune; (4) > 20 ePVS recorded in the basal ganglia (16). Severe CSVD was defined as a CSVD score ≥ 3. Cerebral Amyloid Angiopathy (CAA) was additionally assessed from MRI images using modified Boston Criteria (23). Age was stratified into <80 and ≥80 to align with ICH score usage (24). Hemorrhage volume was analyzed as quartiles and hemorrhage location was categorized as supratentorial vs. infratentorial. Averaged NIHSS scores measured over the first 24 h of admission were collected for secondary analysis and categorized into none (0), mild (1-4), moderate (5-15), moderate–severe (16-20), and severe (21+) neurological deficit (25). Comorbidity burden was defined using the Charlson Comorbidity Index, with severity assessed as Mild (0–2), Moderate (3–4), or Severe (5+) (26).

### 2.3. Statistical analyses

Baseline characteristics are provided as medians and interquartile ranges (IQR) or percentages. Univariable logistic regression was used to assess the individual contributions of major demographic, medication, comorbidities, and clinical and imaging factors. Associations with SDD are reported as crude odds ratios (OR) and 95% confidence intervals (95% CI). Multivariable models were fitted to assess the effects of HD, CSVD, and NIHSS on SDD. Iterative model building was based on a combination of *a priori* determined clinically and statistically significant (*p* < 0.05) factors, which included age, gender, race/ethnicity, antihypertensive, antiplatelet, and anticoagulant treatment, hemorrhage volume, and high systolic blood pressure. Adjusted odds ratios (aOR), and 95% CI are reported from multivariable models.

Structural equation modelling was used to perform mediation analysis according to Baron and Kenny’s method (27), with HD treated as the primary exposure and SDD as the primary outcome. Severe CSVD and stroke severity were independently tested as mediating variables, with stroke severity categorized as a binary variable: Moderate (NIHSS <5) vs. severe (NIHSS ≥5). The proportions of mediated to total effect are reported, along with odds ratios and 95% CI results for each arm of the pathway. Mediation significance was determined through Sobel’s test. All statistical analyses were performed using Stata 16.1 (StataCorp, LLC).

## 3. Results

### 3.1. Cohort demographics

**Figure 1:**
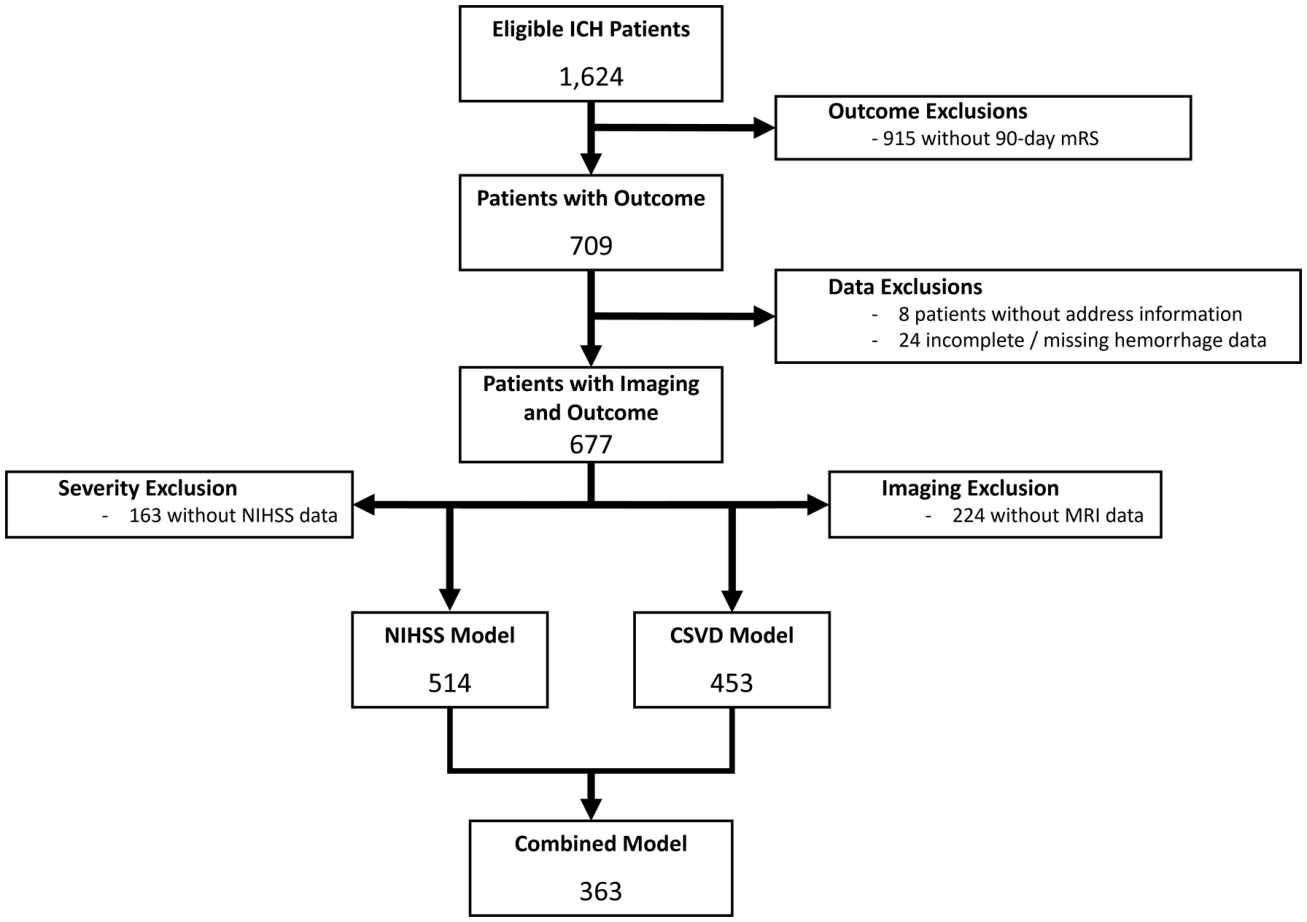
Consort diagram of data exclusions for both NIHSS and CSVD models. The primary reason for data exclusion was lack of available 90-day mRS, followed by missing imaging data and lack of NIHSS assessment.

A total of 1,624 ICH patients were initially identified, 677 of whom were included after excluding patients without 90-day mRS data, complete hemorrhage assessment, and address information (Figure 1), hospitalized between May 2016 and September 2021. The median age was 67 [IQR: 55–77] years, 46.8% were female, and included 43.9% non-Hispanic White, 27.0% non-Hispanic Black, 20.7% Hispanic, 6.1% Asian or Pacific Islander, and 2.4% Other. The median hemorrhage volume was 11.33 [3.39–36.21] cm^3^, with quartiles of: 1^st^ quartile (0–3.39 cm^3^); 2^nd^ quartile (3.39–11.33 cm^3^); 3^rd^ quartile (11.33–36.21 cm^3^); 4^th^ quartile (≥36.21 cm^3^). Overall, the median ADI was 5 (IQR: 2–7), with 167 (24.7%) categorized as HD. Secondary exclusion of patients without MRI imaging and NIHSS scores yielded 514, 453, and 363 patients in the CSVD, NIHSS, and NIHSS-CSVD cohorts, respectively. Exclusion stages and criteria shown in Figure 1.

### 3.2. Univariable associations with SDD

In univariate analysis, the SDD at 90-days after discharge was significantly associated with HD (OR:1.54 [95% CI: 1.06–2.23], *p* = 0.024), severe CSVD (2.14 [1.42–3.21], *p* < 0.001) and moderate (8.03 [2.76–17.15], *p* < 0.001), moderate–severe (32.79 [11.52–93.29], *p* < 0.001), and severe (104.19 [37.66–288.18], *p* < 0.001) NIHSS scores. Older age (1.91 [1.26–2.87], *p* = 0.002), hypertension (2.19 [1.12–4.27]), hemorrhage volumes in the 3^rd^ (4.14 [2.62–6.54], *p* < 0.001) and 4^th^ quartile (14.81 [8.21–26.70], *p* < 0.001; vs. 1^st^ quartile), presence of intraventricular hemorrhage (1.91 [1.21–3.01], *p* < 0.001), infratentorial hemorrhage (vs. supratentorial; 1.91 [1.35–2.70], *p* = 0.005), and low DBP over the first 24 h (2.59 [1.57–4.27], *p* < 0.001) also significantly increased odds of SDD, while patients receiving antihypertensive (0.50 [0.35–0.71], *p* < 0.001), or statin (0.38 [0.27–0.53], *p* < 0.001) treatment showed reduced odds of SDD. Full univariate results are presented in Table 1.

**Table 1:** Univariate associations with severe disability or death.

	No SDD	SDD	Odds ratio [95% CI]	*p*-value
	(*n* = 261)	(*n* = 416)		
Sociodemographics				
Age (≥80)	38 (14.6%)	102 (24.5%)	**1.91 [1.26–2.87]**	**0.002**
Female sex (vs. Male)	113 (43.3%)	204 (49.0%)	1.26 [0.92–1.72]	0.145
Race				
Non-Hispanic White	114 (43.7%)	183 (44.0%)	[Reference]	---
Non-Hispanic Black	64 (24.5%)	119 (28.6%)	1.16 [0.79–1.70]	0.453
Hispanic	66 (25.3%)	74 (17.8%)	0.70 [0.47–1.05]	0.083
Asian	14 (5.4%)	27 (6.5%)	1.20 [0.60–2.39]	0.600
Other/Unspecified	3 (1.2%)	13 (3.1%)	2.70 [0.75–9.68]	0.127
High socioeconomic deprivation	52 (19.9%)	115 (27.6%)	**1.54 [1.06–2.23]**	**0.024**
Marital status (*n* = 660)				
Single	56 (21.8%)	112 (27.8%)	[Reference]	---
Married/Partnered	149 (58.0%)	205 (50.9%)	0.69 [0.47–1.01]	0.056
Widowed/Divorced	52 (20.2%)	86 (21.3%)	0.83 [0.52–1.32]	0.429
Comorbidities				
Hypertension	240 (92.0%)	400 (96.2%)	**2.19 [1.12–4.27]**	**0.022**
Congestive heart failure	68 (26.1%)	134 (32.2%)	1.35 [0.96–1.90]	0.089
Chronic kidney disease	99 (37.9%)	161 (38.7%)	1.03 [0.75–1.42]	0.841
MCID (Medication and ICD)	23 (8.8%)	47 (11.3%)	1.32 [0.78–2.23]	0.302
Diabetes	117 (44.8%)	99 (42.7%)	0.85 [0.62–1.16]	0.310
Atrial fibrillation	45 (20.4%)	170 (40.9%)	1.13 [0.79–1.61]	0.500
Charlson comorbidity index				
Mild (0–2)	68 (26.1%)	71 (17.1%)	[Reference]	---
Moderate (3-4)	60 (23.0%)	94 (22.6%)	1.50 [0.94–2.39]	0.087
Severe (5+)	133 (51.0%)	251 (60.3%)	**1.81 [1.22–2.68]**	**0.003**
Medications				
Antihypertensive	202 (77.4%)	262 (63.0%)	**0.50 [0.35–0.71]**	**0.000**
Antiplatelet	75 (28.7%)	129 (31.0%)	1.11 [0.79–1.56]	0.530
Anticoagulant	77 (29.5%)	150 (36.1%)	1.35 [0.97–1.88]	0.079
Antihyperglycemic	78 (29.9%)	134 (32.2%)	1.11 [0.79–1.56]	0.525
Statin	111 (42.5%)	91 (21.9%)	**0.38 [0.27–0.53]**	**0.000**
Imaging assessment	Hemorrhage volume (Quartile)			
1	106 (40.9%)	64 (15.4%)	[Reference]	---
2	88 (34.0%)	80 (19.2%)	1.51 [0.98–2.32]	0.064
3	48 (18.5%)	120 (28.9%)	**4.14 [2.62–6.54]**	**0.000**
4	17 (6.6%)	152 (36.5%)	**14.81 [8.21–26.70]**	**0.000**
Infratentorial hemorrhage	29 (11.1%)	42 (18.1%)	**1.91 [1.21–3.02]**	**0.005**
Intraventricular hemorrhage	62 (23.8%)	155 (37.3%)	**1.91 [1.35–2.70]**	**0.000**
Cortical Superficial Siderosis (presence) (*n* = 453)	45 (20.2%)	40 (17.3%)	0.83 [0.52–1.33]	0.435
Cerebral Amyloid Angiopathy (Boston Criteria) (*n* = 507)				
Non-CAA	184 (80.0%)	211 (76.2%)	[Reference]	---
CAA possible	35 (15.2%)	52 (18.4%)	1.27 [0.79–2.04]	0.321
CAA probable	11 (4.8%)	15 (5.4%)	1.19 [0.53–2.65]	0.672
Severe CSVD (3+) (n = 453)	52 (23.5%)	92 (39.7%)	**2.14 [1.42–3.21]**	**0.000**
Clinical factors				
High SBP over the first 24 h	5 (1.9%)	20 (4.8%)	2.59 [0.96–6.98]	0.061
Low DBP over the first 24 h	22 (8.4%)	80 (19.2%)	**2.59 [1.57–4.27]**	**0.000**
24-h NIH stroke scale (n = 514)				
None (0)	51 (24.1%)	10 (3.3%)	[Reference]	---
Minor (1–4)	93 (43.9%)	19 (6.3%)	1.05 [0.45–2.41]	0.923
Moderate (5–15)	54 (25.5%)	85 (28.2%)	**8.03 [2.76–17.15]**	**0.000**
Moderate - Severe (16–20)	7 (3.3%)	45 (14.9%)	**32.79 [11.52–93.29]**	**0.000**
Severe (21+)	7 (3.3%)	143 (47.4%)	**104.19 [37.66–288.18]**	**0.000**

Bolded odds ratios and *p*-values indicate significant results.

### 3.3. Stroke severity (NIHSS) multivariable and mediation models

The NIHSS cohort had a median age of 67 (IQR: 55–77) years, were 45.7% female, and included 43.6% non-Hispanic White, 28.2% non-Hispanic Black, 20.1% Hispanic, 6.4% Asian or Pacific Islander, and 1.8% Other. The median hemorrhage volume was 11.09 [3.37–33.01] cm^3^, with quartiles of: 1^st^ quartile (0–3.37 cm^3^); 2^nd^ quartile (3.37–11.09 cm^3^); 3^rd^ quartile (11.09–33.01 cm^3^); 4^th^ quartile (≥33.01 cm^3^). The median ADI value was 5 (IQR: 2–8), with 128 (24.9%) being HD. The median NIHSS was 11 (IQR:2.5–22.5) and 314 (66.3%) had an NIHSS score ≥ 5.

In multivariable modelling, patients showing moderate (aOR: 8.64 [3.55–21.03], *p* < 0.001), moderate–severe (42.82 [12.38–148.10], *p* < 0.001) and severe (90.95 [28.05–294.82], *p* < 0.001) NIHSS score had significantly higher odds of SDD, independent of the effects of hemorrhage volume and other covariates. However, HD was not statistically associated with SDD (1.23 [0.64–2.37], *p* = 0.539). Significantly higher odds of SDD were also found among patients with older age (3.53 [1.77–7.06], *p* < 0.001), severe comorbidity burden on the Charlson Comorbidity Index (2.63 [1.20–5.72], *p* = 0.015), hemorrhage volumes in the 4^th^ quartile (2.57 [1.02–6.45], *p* = 0.045), and infratentorial hemorrhage (2.55 [1.17–5.57], *p* = 0.018). Full results are shown in Table 2.

**Table 2:** Multivariate associates of SDD in separate NIHSS and CSVD models.

	CSVD model (*n* = 453)		NIHSS model (*n* = 514)	
	Adjusted odds ratio [95%CI]	*p*-value	Adjusted odds ratio [95%CI]	*p*-value
Sociodemographics				
Age (≥80)	**2.27 [1.20–4.28]**	**0.011**	**3.53 [1.77–7.06]**	**0.000**
Female gender	0.84 [0.53–1.34]	0.466	0.86 [0.51–1.46]	0.580
Race				
Non-Hispanic White	[Reference]	---	[Reference]	---
Non-Hispanic Black	1.17 [0.66–2.09]	0.589	0.84 [0.43–1.67]	0.625
Hispanic	0.98 [0.53–1.83]	0.952	0.58 [0.28–1.21]	0.145
Asian	1.54 [0.61–3.88]	0.361	1.43 [0.46–4.45]	0.540
Other/Unspecified	3.11 [0.64–15.10]	0.159	2.65 [0.24–29.01]	0.425
High Socioeconomic deprivation	**1.79 [1.04–3.05]**	**0.034**	1.23 [0.64–2.37]	0.539
Comorbidities				
Hypertension	1.92 [0.58–6.28]	0.283	4.21 [0.99–17.85]	0.051
Charlson comorbidity Index				
Mild (0–2)	[Reference]	---	[Reference]	---
Moderate (3-4)	**2.31 [1.10–4.83]**	**0.027**	1.63 [0.68–3.94]	0.275
Severe (5+)	**3.71 [1.91–7.21]**	**0.000**	**2.63 [1.20–5.72]**	**0.015**
Medications				
Antihypertensive	1.03 [0.58–1.81]	0.925	0.48 [0.25–0.93]	0.029
Antiplatelet	0.83 [0.50–1.36]	0.454	0.69 [0.38–1.23]	0.206
Anticoagulant	1.48 [0.92–2.37]	0.109	1.12 [1.20–5.72]	0.694
Imaging assessment	Hemorrhage volume (Quartile)			
1	[Reference]	---	[Reference]	---
2	1.73 [0.97–3.10]	0.063	1.02 [0.51–2.01]	0.959
3	**3.59 [1.96–6.59]**	**0.000**	1.43 [0.69–2.96]	0.330
4	**13.18 [5.90–29.40]**	**0.000**	**2.57 [1.02–6.45]**	**0.045**
Infratentorial hemorrhage	**3.01 [1.58–5.75]**	**0.001**	**2.55 [1.17–5.57]**	**0.018**
Intraventricular hemorrhage	1.36 [0.83–2.22]	0.228	0.91 [0.49–1.68]	0.767
Severe CSVD (3+)	**2.74 [1.67–4.51]**	**0.000**	---	---
Clinical Factors				
High SBP over the first 24 h	2.07 [0.54–7.95]	0.291	2.71 [0.56–13.03]	0.213
Low DBP over the first 24 h	**2.71 [1.31–5.62]**	**0.007**	1.38 [0.56–3.37]	0.481
24-h NIH stroke Scale (*n* = 514)				
None (0)	---	---	[Reference]	---
Minor (1–4)	---	---	0.95 [0.38–2.40]	0.914
Moderate (5–15)	---	---	**8.64 [3.55–21.03]**	**0.000**
Moderate - Severe (16–20)	---	---	**42.82 [12.38–148.10]**	**0.000**
Severe (21+)	---	---	**90.95 [28.05–294.82]**	**0.000**

Bolded odds ratios and *p*-values indicate significant results.

**Figure 2:**
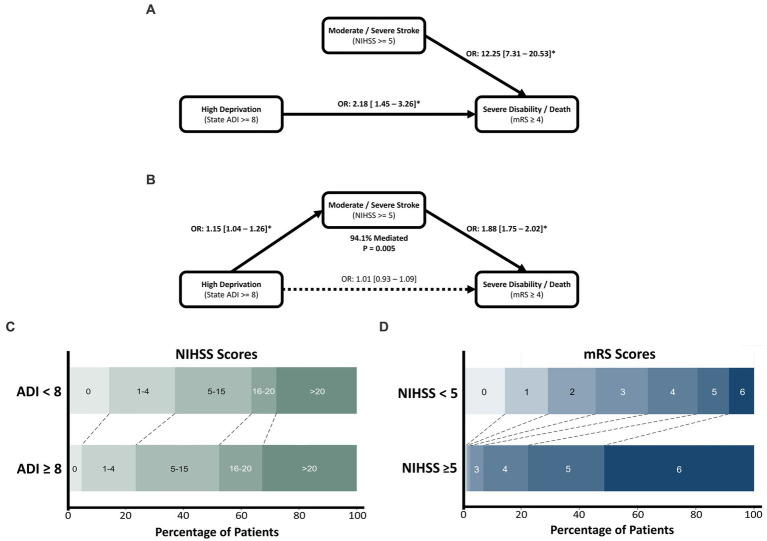
The effect of socioeconomic deprivation on poor functional outcome after ICH, mediated by stroke severity assessed through the NIHSS. Univariate associations of high ADI and NIHSS are shown in **(A)**, with the mediation analysis depicted in **(B)**. Mediation was found to be significant with 94.1% of the effect of deprivation mediated by stroke severity. Mediation was found to be complete with deprivation showing a non-significant direct effect in the SEM model. **(C)** Horizontal stacked bar chart of NIHSS values across deprivation levels. **(D)** Horizontal stacked bar chart of mRS outcomes across stroke severity levels.

In the mediation analyses, HD was significantly associated with higher stroke severity (1.17 [1.04–1.31], *p* = 0.004), which in turn increased the odds of SDD (1.71 [1.56–1.87], *p* < 0.001). Furthermore, HD was not directly associated with SDD independent of the mediation pathway (1.03 [0.93–1.14], *p* = 0.893). Mediation through NIHSS accounted for 94.1% of ADI’s effect on SDD and was found to be statistically significant (*p* = 0.005). Stroke severity was thereby found to ‘completely mediate’ the effect of socioeconomic disadvantage on functional outcome (Figure 2).

### 3.4. CSVD multivariable and mediation model

Among the CSVD cohort, patients had a median age of 65 (IQR: 54–76) years, were 47.9% female, and included 40.7% non-Hispanic White, 28.5% non-Hispanic Black, 21.1% Hispanic, 6.5% Asian or Pacific Islander, and 3.1% Other. The median hemorrhage volume was 8.59 [2.74–21.42] cm^3^, with quartiles of: 1^st^ quartile (0–2.74 cm^3^); 2^nd^ quartile (2.74–8.59 cm^3^); 3^rd^ quartile (8.59–21.42 cm^3^); 4^th^ quartile (≥21.42 cm^3^). The median ADI value was 5 (IQR: 2–7), with 111 (24.5%) being HD. Overall, 146 (31.8%) patients were classified to have severe CSVD (CSVD score ≥ 3).

In the multivariable model, HD (1.79 [1.04–3.05], *p* = 0.034) and severe CSVD (2.74 [1.67–4.51], *p* < 0.001) were independently associated with SDD. Odds of SDD were also increased in patients with older age (2.27 [1.20–4.28], *p* = 0.011), moderate (2.31 [1.10–4.83], *p* = 0.027) and severe (3.71 [1.91–7.21], *p* < 0.001) comorbidity burden, hemorrhage volumes in the 3^rd^ (3.59 [1.96–6.59], *p* < 0.001) and 4^th^ quartiles (13.18 [5.90–5.75], *p* < 0.001), infratentorial hemorrhage (3.01 [1.58–5.75], *p* = 0.001), and low DBP (2.71 [1.31–5.62], *p* = 0.007; Table 2).

**Figure 3:**
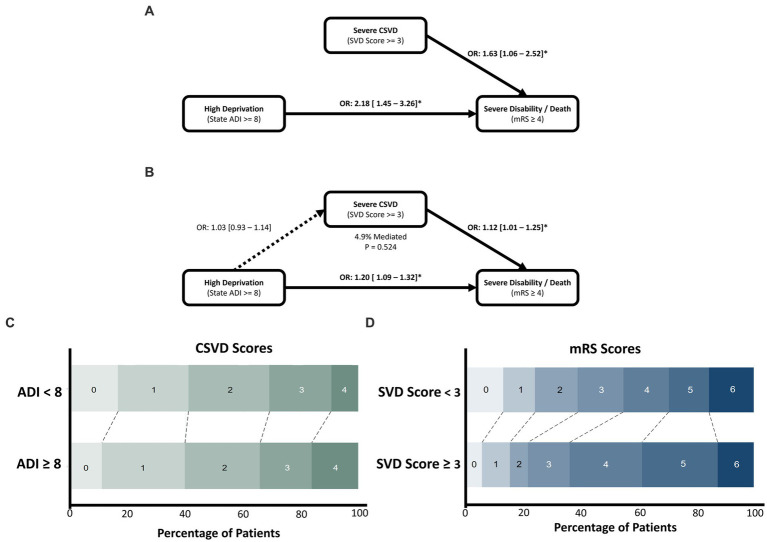
The effect of socioeconomic deprivation on poor functional outcome after ICH, mediated by severe CSVD. Univariate associations of CSVD and deprivation are shown in **(A)**, with the mediation analysis depicted in **(B)**. Both CSVD and deprivation significantly increase odds of experiencing severe disability/death, however CSVD was not associated with deprivation and mediation was non-significant. **(C)** Horizontal stacked bar chart of CSVD score values across deprivation levels. **(D)** Horizontal stacked bar chart of mRS outcomes across SVD score levels.

Mediation modelling found that the direct effects of HD and severe CSVD on SDD were significant (1.12 [1.01–1.25], *p* = 0.031; and 1.20 [1.09–1.32], *p* < 0.001, respectively). However, HD was not significantly associated with severe CSVD (1.03 [0.93–1.14], *p* = 0.524), and mediation was found to be non-significant (*p* = 0.53), accounting for 4.9% of the total effect (Figure 3).

### 3.5. Multivariate associations with SDD in the NIHSS-CSVD model

Among the NIHSS-CSVD cohort, patients had a median age of 65 (IQR: 55–75), were 46.6% female, and included 40.2% non-Hispanic White, 29.8% non-Hispanic Black, 20.4% Hispanic, 7.4% Asian or Pacific Islander, and 2.2% Other. The median hemorrhage volume was 8.63 cm^3^ [2.73–20.86], with quartiles of: 1^st^ quartile (0–2.63 cm^3^); 2^nd^ quartile (2.63–8.63 cm^3^); 3^rd^ quartile (8.63–20.86 cm^3^); 4^th^ quartile (≥20.86 cm^3^). The median ADI was 5 (IQR: 2–8), with 93 (25.6%) being HD. Overall, 110 (30.3%) patients had severe CSVD. The median NIHSS was 7 (IQR:2–17), and 219 (60.3%) had an NIHSS score ≥ 5.

In the combined model, patients with severe CSVD (3.42 [1.75–6.69], *p* < 0.001) and moderate (5.84 [2.27–15.01], *p* < 0.001), moderate–severe (27.59 [7.34–103.69], *p* < 0.001), and severe (36.41 [9.90–133.85], *p* < 0.001) NIHSS scores showed significantly increased odds for SDD, independent of the effects of hemorrhage volume and other covariates. Patients with HD did not (1.26 [0.62–2.57]). Patients with older age (2.71 [1.23–5.94], *p* = 0.013), severe Charlson Comorbidity Index score (2.46 [1.01–6.00], *p* = 0.048), and infratentorial hemorrhage (2.54 [1.10–5.90], *p* = 0.030) additionally showed significantly higher odds for SDD (Table 3).

**Table 3:** Multivariate associates of SDD in a combined NIHSS and CSVD model.

	No SDD (*n* = 183)	SDD (*n* = 180)	Odds ratio (NIHSS Model)	*p*-value
Sociodemographics				
Age (≥80)	24 (13.1%)	39 (21.7%)	**2.71 [1.23–5.94]**	**0.013**
Female gender	84 (45.9%)	85 (47.2%)	0.62 [0.34–1.13]	0.122
Race				
Non-Hispanic White	78 (42.6%)	68 (37.8%)	[Reference]	---
Non-Hispanic Black	47 (25.7%)	61 (33.9%)	0.81 [0.39–1.71]	0.587
Hispanic	44 (24.1%)	30 (16.7%)	0.64 [0.28–1.47]	0.297
Asian	12 (6.6%)	15 (8.3%)	1.12 [0.34–3.63]	0.854
Other/Unspecified	2 (1.1%)	6 (3.3%)	2.82 [0.20–38.82]	0.439
High socioeconomic deprivation	39 (21.3%)	54 (30.0%)	1.26 [0.62–2.57]	0.526
Comorbidities				
Hypertension	167 (91.3%)	177 (98.3%)	3.48 [0.72–16.84]	0.121
Charlson comorbidity index				
Mild (0–2)	46 (25.1%)	17 (9.4%)	[Reference]	---
Moderate (3-4)	46 (25.1%)	43 (23.9%)	1.35 [0.49–3.70]	0.556
Severe (5+)	91 (49.7%)	120 (66.7%)	**2.46 [1.01–6.00]**	**0.048**
Medications				
Antihypertensive	141 (77.1%)	144 (80.0%)	0.57 [0.27–1.22]	0.148
Antiplatelet	55 (30.1%)	51 (28.3%)	0.61 [0.32–1.17]	0.135
Anticoagulant	45 (24.6%)	77 (42.78%)	1.43 [0.78–2.60]	0.243
Imaging assessment	Hemorrhage volume (Quartile)			
1	72 (39.3%)	36 (20.0%)	[Reference]	---
2	62 (33.9%)	41 (22.8%)	1.07 [0.51–2.24]	0.861
3	38 (20.8%)	58 (32.2%)	1.35 [0.60–3.07]	0.471
4	11 (6.0%)	45 (25.0%)	2.55 [0.88–7.41]	0.071
Infratentorial hemorrhage	20 (10.9%)	28 (15.6%)	**2.54 [1.10–5.90]**	**0.030**
Intraventricular hemorrhage	44 (24.0%)	64 (35.6%)	1.04 [0.53–2.06]	0.903
Severe CSVD (3+)	42 (23.0%)	68 (37.8%)	**3.42 [1.75–6.69]**	**0.000**
Clinical factors				
High SBP over the first 24 h	3 (1.6%)	8 (4.4%)	5.54 [0.86–35.65]	0.071
Low DBP over the first 24 h	13 (7.1%)	30 (16.7%)	1.89 [0.68–5.25]	0.223
24-h NIHSS				
None (0)	43 (23.5%)	9 (5.0%)	[Reference]	---
Minor (1–4)	79 (43.2%)	17 (9.4%)	0.75 [0.28–2.07]	0.585
Moderate (5–15)	48 (26.3%)	65 (36.1%)	**5.84 [2.27–15.01]**	**0.000**
Moderate - Severe (16–20)	7 (3.8%)	28 (15.6%)	**27.59 [7.34–103.69]**	**0.000**
Severe (21+)	6 (3.3%)	61 (33.9%)	**36.41 [9.90–133.85]**	**0.000**

Bolded odds ratios and *p*-values indicate significant results.

## 4. Discussion

We report here that ICH patients from HD neighborhoods were more likely to experience SDD independent of CSVD and other major clinical, imaging, and demographic factors. This relationship was strongly mediated by stroke severity and consequently was not apparent in models that controlled for NIHSS. In contrast, no mediating pathway was identified for the effects of HD (on SDD) through CSVD.

### 4.1. Deprivation and CSVD

Evidence from both ischemic and hemorrhage stroke populations highlight CSVD as a major contributor to poor functional outcomes and recurrent/secondary stroke (28). Similarly, socioeconomic status leads to poorer functional outcomes and increased mortality after stroke (3, 29–31). However, the evidence on the relationship between socioeconomic status and CSVD is mixed. Reports have identified accelerated CSVD in individuals with precarious housing (32), and associations have been demonstrated between socioeconomic status and CSVD that break down along racial lines (33). Conversely, other studies show no association between HD and white matter lesions (29, 34). Notably, many of these studies have utilized different markers of CSVD, often choosing to address white matter hyperintensities or aggregate CSVD scores. This may account for some of the variability in findings. It seems unlikely that CSVD is completely unlinked from the effects of socioeconomic deprivation, however. To fully untangle the interplay of CSVD and HD, future studies are encouraged to provide unified models that account for independent CSVD markers across large, heterogenous populations with comprehensive risk assessment. In the meantime, aggregate measures of socioeconomic status and CSVD should be treated as largely independent contributors to ICH outcome.

### 4.2. Deprivation and stroke severity

While no meaningful link was identified between HD and CSVD, we found that patients with HD were more likely to suffer severe stroke and that this disparity is a substantial mechanism through which socioeconomic status leads to poor outcomes. This finding aligns with a previous report that found the link between low income and 3-month post-stroke mortality to be mediated by a scale of consciousness (35). Our work reinforces previous evidence through expanded multivariate models that account for several clinical and demographic confounders in a relatively large sample of ICH patients. We demonstrate an independent effect of socioeconomic status on stroke severity. It is possible that patients within more disadvantaged areas have lower appreciation of early signs of stroke, which results in a delayed hospital presentation, allowing for considerable progression of neurological deficit. It is also likely that HD patients demonstrate hesitancy when calling for emergency transport due to cost concerns or distrust of the medical system (36–38), further exacerbating access to care issues (39).

### 4.3. Limitations

Though our work provides important evidence regarding the roles of socioeconomic status, CSVD, stroke severity, and patient outcomes after ICH, these findings need to be evaluated in the light of following limitations. First, aggregate measures for HD and overall CSVD burden limit the interpretation of the individual social and clinical determinants. Second, while the study population represents a relatively large ICH population, a larger sample size may increase the significance of socioeconomic effects. Additionally, our data, though socio-demographically diverse, represents a single hospital system. We acknowledge that associations between social factors and functional outcomes may be healthcare system driven, and our findings need to be replicated across diverse cohorts. Finally, while over 90% of the effect of HD is mediated through stroke severity, the remaining impact exists without a clear mechanism. Expanded multiple-mediation or structural equation modelling will be needed to provide full characterization of these interactions.

### 4.4. Conclusion

Socioeconomic deprivation contributes to poorer functional outcomes after ICH, with CSVD and stroke severity providing separate possible pathways for this effect. Our results demonstrate a critical link between socioeconomic deprivation and increased stroke severity, leading to SDD at 90 days after discharge. On the contrary, no link was found between deprivation and severe CSVD, which independently impacted patient outcomes. Efforts to reduce admission stroke severity among disadvantaged patients by improving awareness of early stroke symptoms and fostering trust among disadvantaged communities may provide improved outcomes and limit long-term ICH burden.

## Data availability statement

The raw data supporting the conclusions of this article will be made available by the authors, without undue reservation.

## Author contributions

TP and FV conceptualized the study. TP performed data analysis, writing, and data interpretation. JT, AP, and AB performed data collection and contributed to manuscript writing and revision. JT, AP, AB, CJ, EB, HK, CM, TG, RG, VM, DC, and JV performed data collection and retrieval. FV provided project oversight and conceptual guidance. All authors contributed to the article and approved the submitted version.

## Funding

Institutional support was provided by the Houston Methodist Center for Health Data Science and Analytics.

## Conflict of interest

The authors declare that the research was conducted in the absence of any commercial or financial relationships that could be construed as a potential conflict of interest.

## Publisher’s note

All claims expressed in this article are solely those of the authors and do not necessarily represent those of their affiliated organizations, or those of the publisher, the editors and the reviewers. Any product that may be evaluated in this article, or claim that may be made by its manufacturer, is not guaranteed or endorsed by the publisher.

## Abbreviations

ICH: Intracerebral hemorrhage
CSVD: Cerebral small vessel disease
NIHSS: National Institutes of Health Stroke Scale
REINAH: Registry of Neurological Endpoints Assessments among Patients with Ischemic and Hemorrhagic Stroke
ADI: Area deprivation index
HD: High deprivation
mRS: Modified rankin scale
SDD: Severe disability or death
